# Validation practice in health: where do we go from here?

**DOI:** 10.1007/s11136-024-03732-x

**Published:** 2024-07-05

**Authors:** Melanie Hawkins

**Affiliations:** Swinburne University of Technology, Melbourne, VIC Australia

The *Guideline for reporting systematic reviews of outcome measurement instruments (OMIs): PRISMA-COSMIN for OMIs 2024* paper will be a useful checklist resource for journal editors and researchers, particularly for those who are new to reporting systematic reviews. The study has followed an extensive, systematic and comprehensive process to produce this guideline that revises and extends the PRISMA 2020 checklist. The paper and the accompanying guideline are written clearly and in language that is easy to understand despite some of the complex concepts described. But it could be more.

The types-of-validity checklist model of validation (i.e., content/construct/criterion validity) was prominent in education and psychology in the 1970s. It was the validity model that the field of health adopted in the early days of patient-centred measurement. We can consider that this model has served us reasonably well to date. But now, to progress validation practice in health and build on what we know, we need to look to the advances that have occurred in validity theory in education and psychology [1, 2, 6].

Modern validity theorists have iteratively developed their thinking since the 1970s and now understand that the process of validation is an integrated evaluative judgement about *the extent to which a score interpretation is plausible in relation to a specified use in a specific context* [1–4]. This thinking is laid out in the works of many validity theorists, including Samuel J. Messick and Michael T Kane [3, 5], whose works informed the 2014 Standards for Educational and Psychological Testing [1]. The US Food and Drug Administration (FDA) Patient-Focused Drug Development (PFDD) Draft Guidance documents (especially Guidance 3) are now informed by these modern validity theorists, specifically, Kane’s argument-based approach to validation [3, 6]. Guidance 3 (Selecting, Developing, or Modifying Fit-for-Purpose Clinical Outcome Assessments) recommends using Kane’s argument-based approach to generate and evaluate a range of evidence to judge the extent to which Clinical Outcome Assessment (COA) score interpretations are valid for a context of use. As validation practice in health measurement catches up to best practice standards, it is important for authoritative bodies such as PRISMA and COSMIN to be leading the way.

An important area of progress in validation practice is a progressively louder call for the inclusion of evidence based on response processes [1, 7], which is important for understanding the extent to which respondents are engaging with the construct of interest. Response processes evidence is not about item content. It is about the ways in which people think about, interpret, understand and respond to item content in relation to the construct being measured. This evidence is usually generated using qualitative research methods. Response processes evidence is missing from the 2024 PRISMA–COSMIN checklist and the validation literature in health in general 7.

There has also been progress in validation practice regarding fairness in measurement, and careful consideration is required for using score interpretations to make decisions that have social consequences (and, for health measurement, health equity consequences). Patient-reported outcomes (PRO) data are now applied to decision making across a spectrum of uses, from patient treatment to intervention and program development, through to informing health policy. An important intended beneficial consequence of PRO measurement is that of the pursuit of equitable health outcomes, of leaving no one behind. The argument-based approach to validation requires us to examine the assumptions that underpin our interpretation and use of scores and generate evidence to determine if these assumptions are supported in a context or if they prove to be a threat to score interpretation and use in some contexts (e.g., cultural, language, geographical, socioeconomic, across different health conditions). Threats to validity can mean that our measurements lead to decisions that fail to achieve the intended beneficial consequences or, worse, widen the health equity gap.

The PRISMA–COSMIN guideline is a useful document within the bounds of the types-of-validity framework on which it is based. Future iterations of the PRISMA–COSMIN guideline would do well to align with modern validity theory and incorporate an argument-based approach to validation. Rather than reliance on a checklist of types of validity, it is the assumptions underpinning intended score interpretations and use that guide the process of validation, with context being at the forefront of all validity investigations.
